# A phase 1 study of orally administered 5-fluoro-2’-deoxycytidine with tetrahydrouridine in patients with refractory solid tumors

**DOI:** 10.1007/s00280-025-04844-y

**Published:** 2025-12-22

**Authors:** Alice P. Chen, Shivaani Kummar, Larry Rubinstein, Jennifer Zlott, Geraldine O’Sullivan Coyne, Vincent Chung, Edward M. Newman, Heinz-Josef Lenz, Karen Kelly, Liza C. Villaruz, Mariam M. Konaté, Katherine V. Ferry-Galow, Lihua Wang, Robert J. Kinders, Ralph E. Parchment, Joseph M. Covey, Julianne L. Holleran, Richard L. Piekarz, Sarah B. Miller, Lamin Juwara, Jan H. Beumer, James H. Doroshow

**Affiliations:** 1 Division of Cancer Treatment and Diagnosis, National Cancer Institute, NIH, 31 Center Drive, Bldg. 31 Room 3A-44, Bethesda, MD 20892 USA; 2 Department of Medical Oncology and Therapeutics Research, City of Hope National Medical Center, Duarte, CA USA; 3 University of Southern California Norris Comprehensive Cancer Center, Los Angeles, CA USA; 4 University of California Davis Cancer Center, Sacramento, CA USA; 5 University of Pittsburgh Medical Center Hillman Cancer Center, Pittsburgh, PA USA; 6 Clinical Pharmacodynamic Biomarkers Program, Applied/Developmental Research Directorate, Frederick National Laboratory for Cancer Research, Frederick, MD USA; 7 Department of Pharmaceutical Sciences, University of Pittsburgh School of Pharmacy, Pittsburgh, PA USA; 8 Clinical Monitoring Research Program, Clinical Research Directorate, Frederick National Laboratory for Cancer Research, Frederick, MD USA; 9 Center for Cancer Research, National Cancer Institute, Bethesda, MD USA; 10 Present Address: Knight Cancer Institute, Oregon Health and Science University, Portland, OR USA; 11 Present Address: International Association for the Study of Lung Cancer, Denver, CO USA; 12 Present Address: Johns Hopkins University, Baltimore, MD USA

**Keywords:** DNMT1 inhibitors, Cancer epigenetics, Epigenetic modifying agents, Circulating tumor cells, P16

## Abstract

**Purpose:**

The DNA methyltransferase (DNMT) inhibitor 5-fluoro-2’-deoxycytidine (FdCyd) combined with tetrahydrouridine (THU) yielded promising activity in patients with advanced solid tumors, but the intravenous administration schedule of FdCyd limited the clinical feasibility of this treatment program. Therefore, we developed an orally bioavailable formulation of FdCyd and determined the safety, recommended phase 2 dose (RP2D), pharmacokinetics, molecular pharmacodynamic (PD) effects, and antitumor activity of this agent combined with THU.

**Methods:**

Adult patients with advanced solid tumors received FdCyd and THU orally on an intermittent schedule in 21-day cycles; dose levels included once- or twice-daily dosing administered on the first 3–7 days (depending on the dose level) of weeks 1 and 2 of each cycle, with no administration on week 3. Dose escalation followed a standard 3 + 3 design; doses were increased until the target FdCyd maximum plasma concentration corresponding to DNMT inhibition in preclinical studies (1 µM) was reached, after which, the total dose was escalated by increasing the number of days and/or frequency of FdCyd-THU administration. Blood specimens were collected for pharmacokinetic analysis and circulating tumor cell (CTC) PD analyses. Paired pre- and on-treatment (cycle 1 week 3) tumor biopsies were collected during the expansion phase to assess changes in expression of DNMT1 and the epigenetically regulated tumor suppressor protein p16 by immunohistochemistry (IHC), as well as changes in genome-wide DNA promoter methylation.

**Results:**

Fifty-nine patients with solid tumors were enrolled. The RP2D was 160 mg FdCyd once daily combined with 3000 mg THU once daily on days 1–6 and 8–13 of each 21-day cycle. Dose-limiting toxicities (DLT) were grade 3 diarrhea and grade 3 refractory nausea, vomiting, and diarrhea; the most common grade 3–4 adverse events were hematological toxicities. The best response was prolonged stable disease (17 cycles). Active FdCyd plasma concentrations were achieved at doses of 60 mg and higher, and THU exposure was associated with DLT. One of the 7 patients (14%) with analyzable paired tumor biopsy specimens exhibited an appreciable increase in tumor p16 expression, and none had appreciable decreases in qualitative tumor DNMT1 levels. An increase in the proportion of p16-expressing cytokeratin-positive (CK^+^) CTCs was detected in 77% of patients (23 of 30) evaluable for CK^+^ CTC response, while that for vimentin-positive (V^+^) CTCs was 9% of patients (2 of 22) evaluable for V^+^ CTC response. Patients with paired biopsies and a best response of stable disease showed treatment-induced promoter hypomethylation for several epigenetically regulated genes, including tumor suppressor genes.

**Conclusion:**

We determined the RP2D for the combination of orally administered FdCyd and THU and measured prolonged stable disease and tumor suppressor gene hypomethylation in some patients, suggesting potential clinical benefit and molecular activity for this regimen in some patients. The paucity of tumor DNMT1 decreases and p16 re-expression are consistent with the lack of clinical response. However, it may also reflect the timing of on-treatment biopsies (following the 1-week break in FdCyd administration), since increases in p16-expressing CTCs were measured for the majority of CTC-assessable patients.

**Supplementary Information:**

The online version contains supplementary material available at 10.1007/s00280-025-04844-y.

## Introduction

Many cancers harbor aberrant DNA methylation patterns that mediate the silencing of tumor suppressor genes, and targeted therapeutics that inhibit such aberrant DNA methylation continue to be of great interest to the oncology community. DNA methyltransferases (DNMTs) catalyze the methylation of cytosine residues within CpG dinucleotide repeats, which are often found in gene promoters and other regulatory regions. Overexpression of DNMTs in tumor results in promoter hypermethylation and subsequent downregulation of tumor suppressor gene expression [1]. Three DNMT paralogs have been demonstrated to contribute to tumorigenesis: the de novo DNA methyltransferases DNMT3A and DNMT3B, as well as the maintenance methyltransferase DNMT1 [2].

While DNMT inhibition is a well-established therapeutic approach for patients with hematological malignancies, the use of DNA demethylating agents in solid tumor malignancies has remained exploratory. The nucleotide analogs azacytidine and decitabine (5-aza-2’deoxycytidine) are FDA-approved therapies for several leukemias and myelodysplastic syndromes, yielding response rates upwards of 50% in specific indications thereof [3]. Though responses to these agents in patients with solid tumors have been reported, monotherapy response rates for decitabine and azacytidine in this setting are generally low, with substantial hematopoietic toxicity and a correspondingly narrow therapeutic window [4, 5].

The rapid metabolism of nucleoside analog DNMT inhibitors by cytidine deaminase (CDA) substantially limits plasma exposures, and second-generation compounds with improved stability and selectivity have been administered in combination with CDA inhibitors, such as tetrahydrouridine (THU), to substantially prolong plasma half-life [5, 6]. One such second-generation DNMT inhibitor is 5-fluoro-2’-deoxycytidine (FdCyd), a fluoropyrimidine nucleoside analog that has demonstrated antitumor activity in some patients with solid tumors when administered intravenously (IV) in combination with THU in early-phase clinical testing [7, 8]. Modest response rates were measured for the IV-administered combination, with one caveat being the onerous administration schedule (ten 3-hour infusions each month) that may have limited patient participation [8]. As such, we developed an orally bioavailable formulation of FdCyd and demonstrated that this formulation, in combination with orally administered THU, yields plasma concentrations sufficient for inhibition of DNA methylation and CDA, respectively, in cynomolgus monkeys, with comparable plasma concentrations achieved in a pilot pharmacokinetic (PK) study in humans [9]. Here, we present safety, PK, pharmacodynamic (PD), and response data for patients with solid tumors enrolled in a phase 1 study of the orally administered FdCyd + THU combination.

## Patients and methods

### Eligibility criteria

The study enrolled patients 18 years and older with histologically confirmed solid tumors for which standard therapies had been ineffective, or for which standard therapies did not exist. Patients were required to have measurable or evaluable disease, a life expectancy of > 3 months, and a Karnofsky performance status of ≥ 60%. Adequate organ and marrow function were also required, as defined by absolute neutrophil count ≥ 1,500/µL, platelet count ≥ 100,000/µL, total bilirubin < 1.5× the institutional upper limit of normal (ULN), alanine aminotransferase and/or aspartate aminotransferase ≤ 3× ULN (or ≤ 5× ULN for patients with liver metastases), and creatinine < 1.5× ULN (or creatinine clearance of ≥ 60 mL/min for patients with levels ≥ 1.5× ULN). Patients were required to have completed any prior therapies ≥ 4 weeks prior to enrollment and must have recovered to eligibility levels for performance status and organ function following any prior toxicities. Patients were also required to be willing to provide blood and urine samples. For patients enrolled on the expansion cohort, individuals were required to have tumors amenable to biopsy and be willing to undergo a tumor biopsy; have tumor tissue collected during a medically necessitated procedure; or provide archival tumor biopsy tissue. Patients excluded from this study were those with known brain metastases or carcinomatous meningitis (with the exception of patients whose brain metastatic disease status had remained stable for ≥ 2 months after treatment of the brain metastases) or with clinically significant illnesses that would compromise participation in the study, including active or uncontrolled infection, immune deficiencies, known HIV infection requiring protease inhibitor therapy, hepatitis B, hepatitis C, uncontrolled diabetes, uncontrolled hypertension, symptomatic congestive heart failure, unstable angina pectoris, myocardial infarction within the past 6 months, uncontrolled cardiac arrhythmia, or psychiatric illness/social situations that would limit compliance with study requirements.

### Trial design

This multicenter study (ClinicalTrials.gov identifier: NCT01534598) was conducted under an NCI-held investigational new drug application, and institutional review board approval was obtained at each participating site. Informed consent was obtained from all individual participants included in the study.

FdCyd and THU were supplied by the NCI Division of Cancer Treatment and Diagnosis. Patients were administered both drugs orally in 21-day cycles, and dose escalation proceeded according to a traditional 3 + 3 design as per the dose escalation schema shown in Supplementary Table S1. Per protocol and based on preclinical data indicating inhibition of DNA methylation in vitro at FdCyd concentrations ≥ 1 µM (245 ng/mL) [10], dose escalation proceeded until the target C_max_ of 1 µM FdCyd was reached in at least 1 patient. After this target C_max_ value was achieved (DL5) and in the absence of dose-limiting toxicities (DLT), the total dose was then escalated by increasing the number of days of FdCyd-THU administration per week (DL6-9) and then by changing from once daily (QD) to twice daily (BID) administration (DL10-11). Patients maintained a study diary to note any side effects or concurrent medications. The NCI Common Terminology Criteria for Adverse Events version 4.0 was used to grade adverse events. Tumor response was assessed by radiography at baseline and every 2 cycles thereafter and evaluated per RECIST version 1.1 [11]. Following determination of the maximum tolerated dose (MTD)/recommended phase 2 dose (RP2D), an additional 13 patients were enrolled on a RP2D expansion cohort for assessments of pharmacodynamic biomarkers in baseline and on-treatment tumor biopsies. The timepoint for the on-treatment biopsy (Cycle 1 Week 3 [C1W3]) was selected to optimize for detection of FdCyd-mediated tumor epigenetic changes, as previous blood-based measurements of FdCyd-induced gene expression changes in clinical studies of intravenously administered FdCyd demonstrated that such changes often occur only after multiple days or weeks of FdCyd therapy [7, 8].

### Dose-limiting toxicity definitions and dose modification criteria

DLTs were defined as toxicities that were at least possibly related to FdCyd or THU within the first cycle of treatment and met one of the following criteria: grade ≥ 3 non-hematological toxicity (including grade 3 nausea, vomiting, or diarrhea only if refractory to standard supportive care; grade 3 electrolyte toxicities only if unable to be corrected to ≤ grade 1 within 24 h; and tumor pain only if refractory to analgesics); grade 4 thrombocytopenia; or grade 4 neutropenia lasting ≥ 5 days or febrile neutropenia. Dose reductions were made in response to grade 3–4 drug-related non-hematological toxicities (with the exceptions of tumor pain, nausea, vomiting, and diarrhea unless refractory to supportive measures; alopecia; and electrolyte abnormalities resolving to ≤ grade 1 within 24 h with supportive care) and grade 4 hematological toxicities (with the exceptions of anemia, lymphopenia, and leukopenia in the absence of neutropenia). In response to the aforementioned toxicities, FdCyd and THU were held until the toxicities were resolved to ≤ grade 2 and then resumed at the next-lower dose level.

### Pharmacokinetics

Blood specimens for PK analysis were collected on Cycle 1 Day 1 (C1D1) prior to THU administration and then at 15, 30, and 45 min and 1, 2, 3, 4, 5, and 6 h after THU administration, as well as on C1D2 and C1D3 prior to and 2 h after THU administration. Urine specimens for PK analysis were collected on C1D1 prior to treatment and then at every void from 0 to 24 h after THU administration. All specimens for PK analysis were processed and analyzed by LC-MS/MS as described previously [7, 12], and Phoenix WinNonlin software was used for data analysis.

### Protein pharmacodynamic biomarker analysis of tumor biopsy specimens

Core needle tumor biopsy specimens were collected at baseline (C1D1 pre-dose) and during C1W3 and flash-frozen as previously described [13]. For qualitative DNMT1 and p16 immunohistochemistry (IHC) analyses, frozen cores were thawed into formalin, and formalin-fixed, paraffin-embedded (FFPE) sections were stained using EPR3521(2) rabbit monoclonal antibody (Abcam) or BOND Ready-To-Use Primary Antibody p16 (6H12; Leica), respectively, together with the Leica BOND Polymer Refine Detection kit. Quantitative immunofluorescence microscopy analysis of pHH3-expressing cells was performed as described previously [13].

### CellSearch^®^ analysis of CTC p16 expression in patient blood specimens

Each blood specimen for CTC analysis (7.5 mL) was collected into a 10-mL CellSave preservative tube (Menarini Silicon Biosystems). Specimens were collected pre-treatment and then 6 h after THU administration on C1D1; prior to THU administration on C1D2 and either C1D8 or C1D9 (at the end of the first round of drug administration); on day 1 of cycle 2 and all subsequent cycles; and optionally at time of progression. Blood sample collection for CTC analysis was performed relative to the morning dose of THU for patients on the BID dose levels (DL10-11). CTC samples were processed and analyzed for p16, cytokeratin, and vimentin as described previously [8]. Serial baseline blood specimens (C1D1 pre, C1D1 post, and C1D2) were obtained in light of baseline variability in CTC counts and p16 expression, and patients were considered assessable for cytokeratin-positive (CK^+^) CTC p16 response if at least one of these baseline specimens (i.e., C1D1 pre, C1D1 post, and/or C1D2) and at least one post-treatment specimen (collected C2D1 or later) each contained ≥ 6 CTCs, as described [8]. For patients with multiple baseline specimens, the specimen with the highest frequency of p16-positive CTCs was considered “baseline,” as described [8].

### Genome-wide DNA methylation sequencing analysis

Tumor biopsy cores collected at baseline and C1W3 underwent nucleic acid extraction using the Qiagen AllPrep FFPE Nucleic acid Extraction kit and QiaCube automated platform. Extractions were performed on 1–2 biopsy cores per patient per time point; for specimens with low tumor content, tumor microdissection was performed prior to extraction. Genome-wide DNA methylation sequencing on the isolated DNA was performed by Zymo Research using Methyl-MiniSeq Genome-Wide Bisulfite Sequencing methodology. Sequence reads from bisulfite-treated Classic RRBS libraries were identified using standard Illumina base calling software. Raw FASTQ files were trimmed of adapter, filled-in nucleotides, and low-quality base calls using TrimGalore 0.6.4. FastQC 0.11.8 was used to assess the effect of trimming and overall quality distributions of the data. Alignment to the hg19 reference genome was performed using Bismark 0.19.0. Methylated and unmethylated read totals for each CpG site were called using Bismark Methylation Extractor. The methylation level of each sampled cytosine was estimated as the number of reads reporting a C, divided by the total number of reads reporting a C or T.

### Statistical and bioinformatics analyses

All statistical analyses were performed in R. Differentially methylated CpG sites (DMCs)—both hypo- and hyper-methylated—were identified with the R package DSS [14] using the two-sided Wald test for beta-binomial distributions with smoothing step as recommended. Pairwise comparisons were conducted between pre-treatment and on-treatment samples at C1W3. A CpG site was considered significantly differentially methylated if it had FDR < 0.05 and a methylation percentage difference ≥ 10% between the paired samples. Regions with many significant DMCs were detected as differentially methylated regions (DMRs) using DSS function *callDMR*. The annotation analysis package annotatr [15] was used to obtain the genomic context (e.g., mapping to promoters) for each DMR.

Correlations between gene expression and methylation level in the GDSC-MGH-Sanger dataset (*n* = 417 cell lines) were evaluated with CellMiner CDB [16]. Genes with Pearson *r* ≤ −0.3 were considered likely epigenetically regulated.

Gene set enrichment analysis was conducted with Enrichr [17–19], and the results were extracted for gene ontology biological processes and molecular functions.

## Results

### Patient demographics

A total of 59 patients were enrolled on this study from March 2012 to August 2019 (Table 1). The median patient age was 60 years (range: 32–85 years), and the median number of prior therapies was 5 (range: 0–19). Numerous tumor types were represented, with the most prevalent being colorectal and head and neck and carcinomas (14 and 12 patients, respectively).

**Table 1:** Patient characteristics

Characteristics	Number of patients
Number of patients, total Female Male	59 29 30
Median Age, y (range)	60 (32–85)
Karnofsky performance status (%)	
100	6
90	16
80	27
70	5
60 40	4 1
*Diagnosis*	
Colorectal carcinoma Head and neck Genitourinary Breast Lung NSCLC SCLC Lung NOS Upper gastrointestinal tumors Hepatocellular carcinoma Pancreatic Esophageal Cervical Mesothelioma Ovarian Melanoma Sarcoma Appendiceal Neuroendocrine Anal	14 12 7 5 4 1 1 2 2 1 2 2 1 1 1 1 1 1
Median number of prior therapies (range)	5 (0–19)

### Toxicity

The combination of oral FdCyd and THU was well-tolerated. The maximum tolerated dose was found to be 160 mg FdCyd administered orally once daily + 3000 mg THU administered orally once daily on days 1–6 and 8–13 of each 21-day cycle (dose level 8). As observed in the phase 1 study of the intravenously administered combination [7], the most commonly occurring drug-related grade 3/4 adverse events for the orally administered combination were hematological toxicities, followed by gastrointestinal toxicities (Tables 2 and 3). The twice-daily THU dosing schedule (dose levels 10 and 11) yielded greater toxicity than the single THU dose administered on lower total dose levels (Tables 2 and 3). Two patients had DLTs on DL9 (the highest once-daily dose level): 1 patient with grade 3 refractory nausea, vomiting, and diarrhea, and 1 with grade 3 diarrhea (Supplementary Table S2). Although no DLTs were measured in the initial 6 patients accrued to the lowest twice-daily dose level, DL10 (3 were inevaluable), 2 patients had DLTs (grade 3 nausea, vomiting, and diarrhea, and grade 4 thrombocytopenia) on DL11, which was the maximum administered dose. Accrual continued at DL10 but was halted after both of the accrued patients experienced DLTs: grade 3 oral mucositis and grade 4 neutropenia. These DLTs on DL10 and DL11 suggest that extending FdCyd exposure to the second 12-hour period each day may increase the likelihood of high-grade toxicities, even with lower C_max_ and total exposure relative to the 160 mg daily administered at DL8. Following accrual of an additional 6 patients to DL8 and the resulting absence of any DLTs (Supplementary Table S2), DL8 was determined to be the MTD and RP2D. The expansion cohort patients enrolled on DL8 were also included in the toxicity analyses for this study (Tables 2 and 3, Supplementary Table S2).

**Table 2:** Adverse events attributed to oral FdCyd + THU

Adverse event	Grade 3	Grade 4
*Hematological*		
Anemia	5 (8%)	–
Leukopenia	2 (3%)	3 (5%)
Lymphopenia	11 (19%)	3 (5%)
Neutropenia	1 (2%)	3 (5%)
Thrombocytopenia	1 (2%)	1 (2%)
*Gastrointestinal*		
Diarrhea	5 (8%)	–
Mucositis oral	2 (3%)	–
Nausea	3 (5%)	–
Vomiting	2 (3%)	–
*Metabolism and nutrition disorders*		
Dehydration	1 (2%)	–
Hypokalemia	3 (5%)	–
Hyponatremia	2 (3%)	–
Hypophosphatemia	3 (5%)	–
*Liver function*		
Alanine aminotransferase increased	1 (2%)	–
Aspartate aminotransferase increased	1 (2%)	–
*Other*		
Fatigue	2 (3%)	–

Worst-grade events (≥ grade 3) for each patient that were at least possibly related to FdCyd + THU treatment are shown. The percentage of patients experiencing each event is shown in parentheses (*n* = 59 patients)

**Table 3:** Adverse events attributed to oral FdCyd + THU

Adverse event	*Once-daily THU* (*n* = 48)		*Twice-daily THU* (*n* = 11)	
	Grade 3	Grade 4	Grade 3	Grade 4
*Hematological*				
Anemia	1 (2%)	–	4 (36%)	–
Leukopenia	–	–	2 (18%)	3 (27%)
Lymphopenia	9 (19%)	1 (2%)	2 (18%)	2 (18%)
Neutropenia	–	–	1 (9%)	3 (27%)
Thrombocytopenia	–	–	1 (9%)	1 (9%)
*Gastrointestinal*				
Diarrhea	2 (4%)	–	3 (27%)	–
Mucositis oral	1 (2%)	–	1 (9%)	–
Nausea	2 (4%)	–	1 (9%)	–
Vomiting	1 (2%)	–	1 (9%)	–
*Metabolism and nutrition disorders*				
Dehydration	1 (2%)	–	–	–
Hypokalemia	1 (2%)	–	2 (18%)	–
Hyponatremia	1 (2%)	–	1 (9%)	–
Hypophosphatemia	1 (2%)	–	2 (18%)	–
*Liver function*				
Alanine aminotransferase increased	1 (2%)	–	–	–
Aspartate aminotransferase increased	1 (2%)	–	–	–
*Other*				
Fatigue	1 (2%)	–	–	–

Worst-grade events (≥ grade 3) for each patient that are at least possibly related to FdCyd + THU treatment are shown. The percentage of patients experiencing each event is shown in parentheses for each THU dosing schedule (*n* = 48 and *n* = 11 for patients receiving once-daily and twice-daily THU, respectively)

### Clinical response

**Fig. 1:**
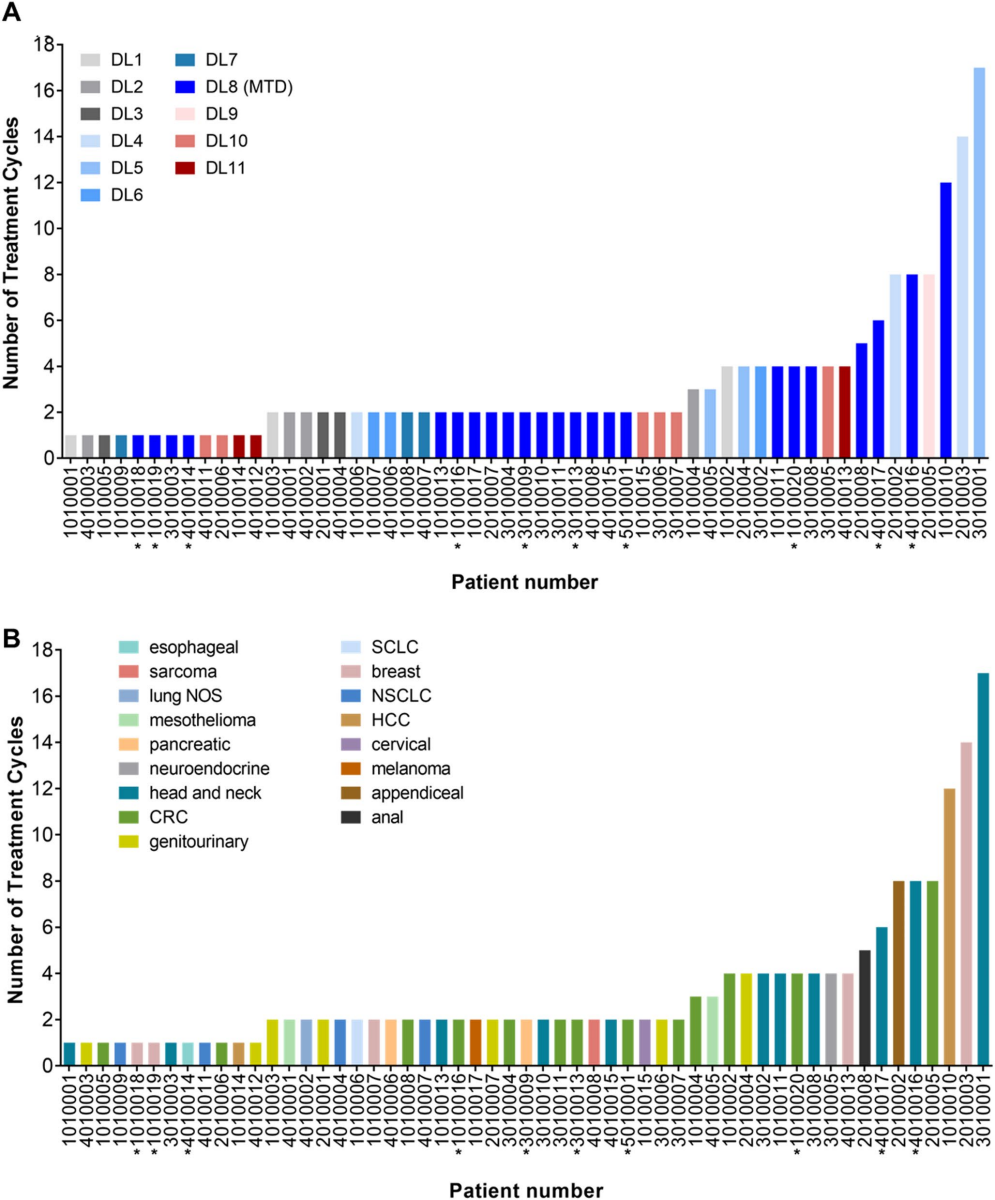
Clinical response to oral FdCyd + THU therapy. The number of cycles of treatment completed is shown for each patient on study for > 1 cycle, colored according to starting dose level (**A**) or tumor type (**B**). The best response measured on this study was stable disease. Patients who were biopsied for tumor PD analysis are indicated by asterisks

The best response to the oral FdCyd-THU combination was prolonged stable disease (Fig. 1). Six patients experienced stable disease for ≥ 8 cycles: 1 patient with head and neck squamous cell carcinoma (HNSCC) on DL5 who was on study for 17 cycles; 2 patients on DL4 (with breast and appendiceal carcinoma and on study for 14 and 8 cycles, respectively); 1 patient with colorectal carcinoma (CRC; 8 cycles) who started on DL9 and was then dose-reduced to DL8 and subsequently DL7 due to grade 3 anemia, abdominal pain, nausea, vomiting, diarrhea, and dehydration during cycle 1 and grade 3 dehydration during cycle 2, respectively; and 2 patients on the MTD/RP2D dose level (DL8), 1 with HCC and 1 with HNSCC on study for 12 and 8 cycles, respectively (Fig. 1).

### Pharmacokinetic analysis

**Fig. 2:**
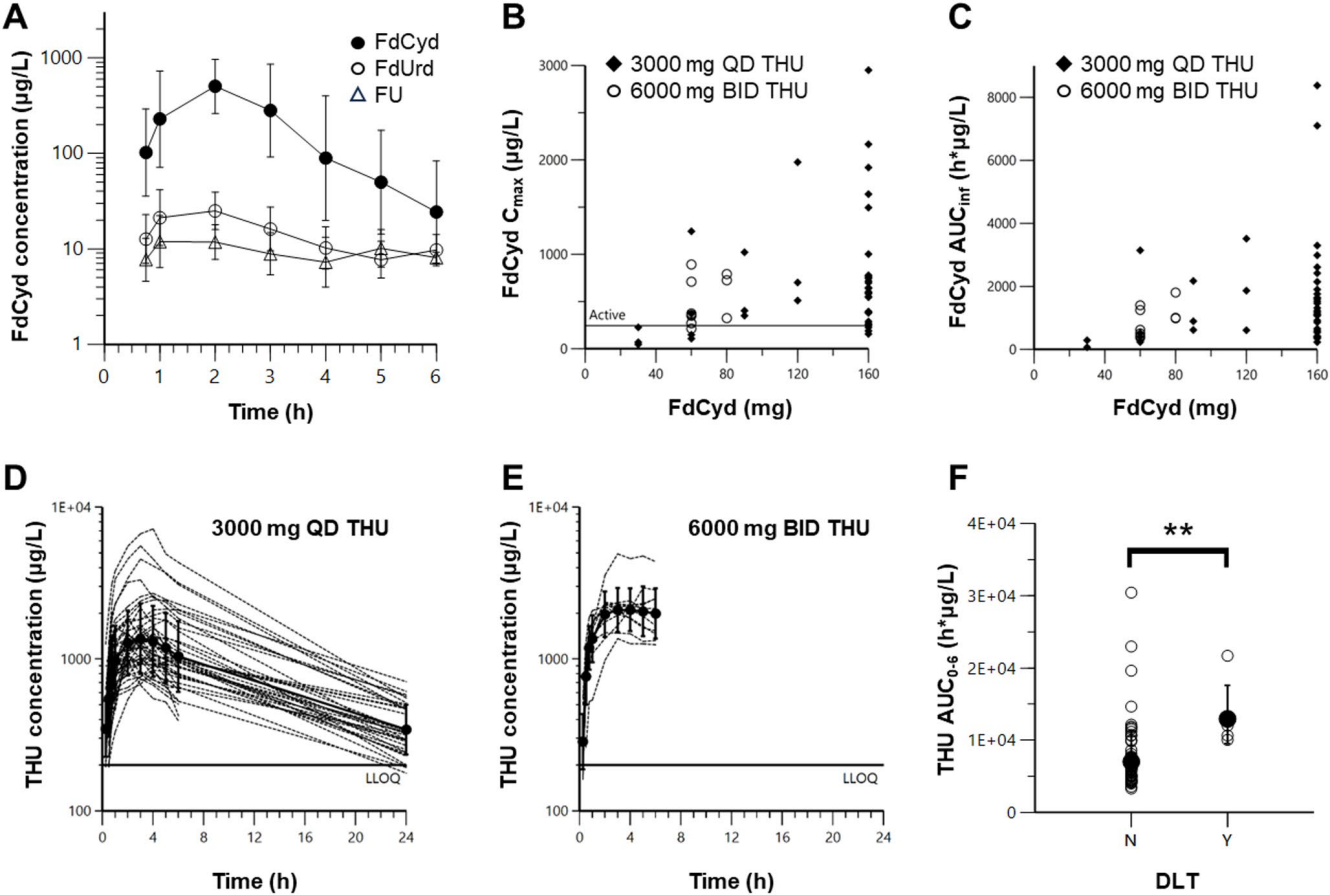
**P**lasma pharmacokinetic analysis of oral FdCyd combined with THU.** A**, Geometric mean plasma concentrations of FdCyd (⬤), FdUrd (), and FU (ρ) for patients receiving 160 mg FdCyd + 3000 mg THU (dose levels 5–9). Error bars indicate standard deviation; *n* = 31 patients. **B**,** C**, FdCyd exposure (C_max_ [**B**] and AUC [**C**]) as a function of FdCyd and THU dose. Active target exposure of 245 ng/mL (1 µM) is indicated by thick horizontal black lines. **D**,** E**, Plasma concentrations of THU for patients receiving THU on a 3000 mg once daily (QD; **D**) or 6000 mg twice daily (BID; **E**) schedule. Plasma THU pharmacokinetic profiles for individual patients (dashed lines), along with geometric means (solid lines and circles), are shown; error bars indicate geometric standard deviations. **F**, THU exposure-toxicity relationship following oral FdCyd + THU administration. Closed circles represent geometric mean AUC_0 − 6_ values, while open circles indicate values for individual patients; error bars indicate geometric standard deviations. Samples with values below the lower limit of quantitation (200 ng/mL) were imputed as 100 ng/mL. Statistical significance (*P* = 0.007) is indicated by asterisks

Plasma pharmacokinetic analysis demonstrated that FdCyd concentrations peaked at approximately 1.2 h post-dose, with an elimination half-life of 1.5 h (Fig. 2A, Supplementary Table S3). FdCyd exhibited a largely dose-proportional exposure profile, though with substantial interpatient variability (Fig. 2B-C), and the target C_max_ of 1 µM (245 ng/mL) [28] was reached by individual patients starting at 60 mg (Fig. 2B, Supplementary Fig. S1). Dose-normalized FdUrd concentrations at 2 h did not rise over days 1–3, but were higher with 6000 mg THU twice daily relative to 3000 mg THU once daily (Supplementary Fig. S2B, Supplementary Table S4). THU concentrations peaked at around 3 h post-administration and had a half-life of 8.7 h (Fig. 2D, E; Supplementary Table S5). Interestingly, the doubling of both THU dose (3000 to 6000 mg) and frequency (once to twice daily) resulted in an approximate 10-fold increase in 24 and 48 h trough concentrations as opposed to the expected 4-fold increase, possibly due to saturable and more prolonged absorption, consistent with the decreased dose-normalized C_max_ and AUC_0 − 6_ as dose and schedule were intensified (Supplementary Table S5). The more intense THU exposure appeared to increase the dose-normalized FdCyd C_max_ (*P* = 0.028; Supplementary Table S3; Supplementary Fig. S2A), although this effect could not be detected in the apparent clearance (*P* = 0.16; Supplementary Table S3). As noted above, 2 h FdCyd concentrations were also increased with the higher dose of THU. Surprisingly, at the higher THU dose, dose-normalized FdUrd C_max_ (*P* = 0.0016) and AUC (*P* = 0.028) also increased. The FdUrd/FdCyd metabolic ratios of both C_max_ (*P* = 0.93) and AUC (*P* = 0.82) were not impacted by the increase in THU (Supplementary Table S4). This suggests that additional THU increases the oral bioavailability of FdCyd but does not further reduce subsequent systemic cytidine deaminase-mediated metabolic conversion of FdCyd to FdUrd. Urine excretion data, though limited and at very low concentrations, also suggested increased FdCyd and FdUrd excretion with higher THU dosing (Supplementary Table S6). While there was no statistically significant relationship between week 1 cumulative FdCyd exposure and occurrence of DLT (Supplementary Fig. S3), patients who experienced a DLT had significantly higher THU exposures than those who did not (Fig. 2F); this difference remained statistically significant regardless of whether THU exposure was expressed as AUC_0 − 6_ (*P* = 0.007), C_max_ (*P* = 0.005), C_24h_ (*P* = 0.002), or C_48h_ (*P* = 0.004).

### Pharmacodynamic analyses

#### Analysis of DNMT1 levels and cell cycle arrest in tumor biopsy specimens

Because covalent trapping of DNMT1 on DNA has been shown to induce DNMT proteasomal degradation [20, 21], we assessed whether oral FdCyd-THU therapy yielded changes in tumor DNMT1 levels via IHC analysis of baseline (pre-dose) and on-treatment (Cycle 1 Week 3; C1W3) biopsy specimens. Of the 7 patients with assessable paired biopsies, none showed appreciable treatment-induced decreases in DNMT1 levels; indeed, 2 patients (5010001 and 1010020, with best responses of PD and 4 cycles SD, respectively) demonstrated increases in DNMT1 levels following FdCyd-THU administration (Supplementary Table S7; Supplementary Fig. S4), consistent with DNMT rebound effects reported for other DNMT-inhibiting agents [22, 23].

DNMT inhibition has also been shown to result in DNA damage and G_2_/M phase cell cycle arrest [24, 25], and we therefore assessed changes in tumor levels of the mitotic marker serine 10–phosphorylated histone H3 (pHH3) using a previously validated quantitative immunofluorescence microscopy assay for pHH3 [13] in baseline and C1W3 biopsies. Among the 6 patients with assessable paired tumor specimens, there were no appreciable changes in the percentage of pHH3^+^ cells in response to FdCyd-THU (Supplementary Fig. S5). This lack of treatment-induced reduction in pHH3 is consistent with the paucity of clinical activity for oral FdCyd-THU therapy.

#### P16 expression in tumor tissue and circulating tumor cells

Because p16 has been demonstrated to be a pharmacodynamic biomarker of FdCyd epigenetic effects [8], we assessed treatment-associated p16 modulation in both tumor and CTCs. Eight patients had baseline and/or on-treatment tumor tissue available for p16 IHC evaluation; tumors from all these patients had some level of p16 expression at baseline (Table 4). Of the 7 patients with paired biopsies of sufficient quality for analysis, just 2 showed qualitative modulation of tumor p16 staining in response to FdCyd-THU treatment: patient 1,010,019 exhibited a slight increase in p16 expression, while patient 1,010,020 exhibited a slight decrease (Table 4; Supplementary Fig. S6). The overall lack of substantial tumor p16 expression modulation is consistent with the general lack of antitumor activity of this regimen in these patients.

**Table 4:**
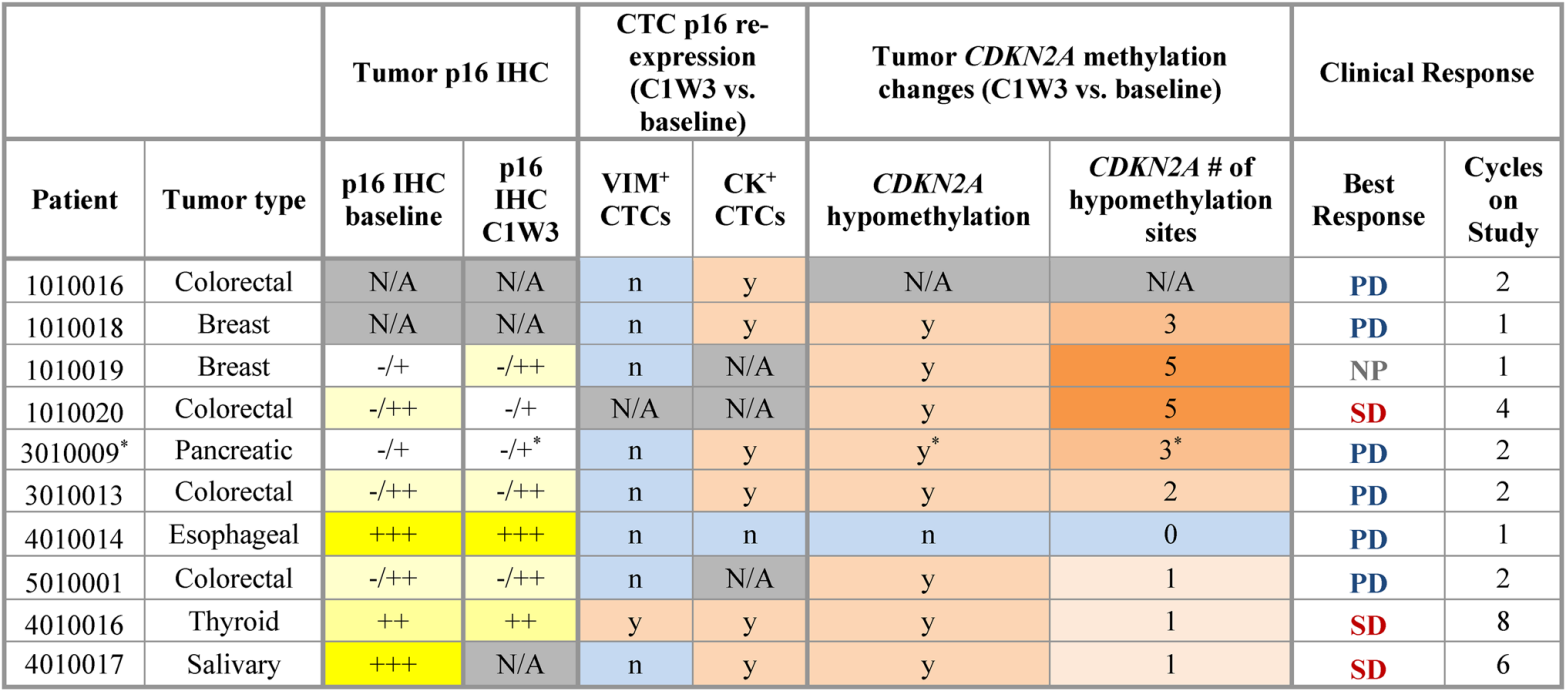
Therapy-associated changes in tumor and CTC p16 expression in expansion cohort patients receiving oral FdCyd + THU

N/A, not available (gray); n, no (blue); y, yes (orange); NP, not assessed per protocol; PD, progressive disease; SD, stable disease. For tumor p16 IHC, staining intensity is indicated by yellow shading, from low and heterogenous staining intensity (-/+) to moderate heterogenous staining (-/++), moderate homogenous staining (++), and high homogenous staining (+++). *The on-treatment biopsy for patient 3,010,009 was collected during cycle 2 week 3 (C2W3)

**Table 5:** Epigenetically regulated genes with FdCyd-THU–induced promoter hypomethylation in patients with a best response of stable disease

Patient	Gene	Chr.	Gene name (full)		Tumor suppressor activity ref.
**4,010,016**	BMPR1B	chr4	Bone morphogenetic protein receptor type-1B		[30]
	CDH1	chr16	Cadherin-1		[31]
	FCGRT	chr19	IgG receptor FcRn large subunit p51		[32]
	GSTM1	chr1	Glutathione S-transferase Mu 1		
	GYG2	chrX	Glycogenin-2		
	INAVA	chr1	Innate immunity activator protein		
	LRIG3	chr12	Leucine-rich repeats and Ig-like domains protein 3		[33]
	MAMDC2	chr9	MAM domain-containing protein 2		[34]
	TM4SF19	chr3	Transmembrane 4 L6 family member 19		
	WDR86	chr7	WD repeat-containing protein 86		
**4,010,017**	ACVR1	chr2	Activin receptor type-1		
	GPRIN1	chr5	G protein-regulated inducer of neurite outgrowth 1		
	IFI16	chr1	Gamma-interferon-inducible protein 16		
	MPV17L	chr16	Mpv17-like protein		
	PLS3	chrX	Plastin-3		
	RASSF1	chr3	Ras association domain-containing protein 1		[35]
	RPS6KB1	chr17	Ribosomal protein S6 kinase beta-1		
	TET1	chr10	Methylcytosine dioxygenase TET1		[36]
	TTC19	chr17	Tetratricopeptide repeat protein 19, mitochondrial		
	UHRF1	chr19	E3 ubiquitin-protein ligase UHRF1		
**Patient**	**Gene set name**			**Number of genes**	**Adj. P-value**
**1,010,020**	Transcription regulator activity			39	1.32e^− 12^
	Sequence-specific DNA binding			27	8.48e^− 8^
	DNA binding/transcription activation			26	9.07e^− 8^

*Top*: All genes shown for patients 4,010,016 and 4,010,017 are those that are likely epigenetically regulated (i.e., genes for which RNA expression is negatively correlated with DNA promoter methylation across NCI-60 cell lines based on CellMinerCDB data and analysis tools [44])

*Bottom*: Top 3 gene ontology molecular function (GOMF) pathway gene sets with significant FdCyd-induced promoter hypomethylation for patient 1,010,020. For each pathway, the number of likely epigenetically regulated genes (defined as those for which RNA expression is negatively correlated with DNA promoter methylation across GDSC cell lines in CellMinerCDB [16]) that exhibit treatment-induced hypomethylation and the corresponding FDR-adjusted p-value are shown

Chr., chromosome; Ig, immunoglobulin; ref., reference

To assess the effects of FdCyd on the epigenetic regulation of p16, we also assessed tumor promoter methylation of the p16-encoding gene *CDKN2A*, as measured in our genome-wide promoter hypomethylation analysis of tumor biopsy cores. A total of 6 expansion cohort patients had both reportable tumor *CDKN2A* hypomethylation data and qualitative tumor p16 IHC data available. Five of these 6 patients exhibited treatment-associated decreases in tumor *CDKN2A* methylation, yet only 1 showed increased tumor p16 expression by IHC (Table 4), indicating a potential discrepancy between the changes in transcriptional versus translational level regulation of tumor p16 expression at the examined timepoints.

**Fig. 3:**
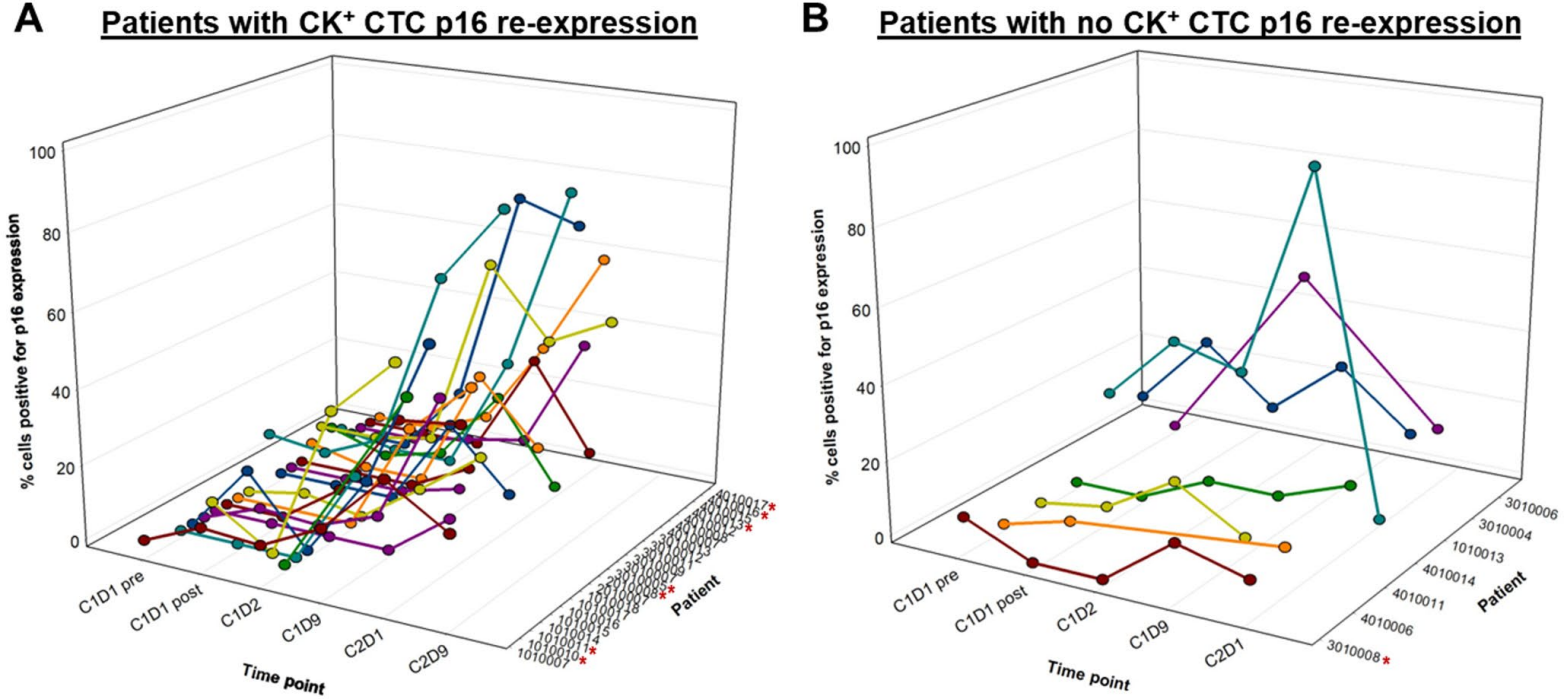
P16 re-expression in CK^+^ CTCs following oral FdCyd + THU therapy. The % CK^+^ CTCs positive for p16 over time is shown for patients in which FdCyd-induced CK^+^ CTC p16 re-expression was measured (**A**) and those for whom no such p16 re-expression was observed (**B**). Red asterisks indicate patients with a best response of stable disease. Data are shown for specimens collected during the first 2 cycles of treatment

A total of 30 patients were evaluable for assessment of FdCyd-induced changes in the proportion of p16-expressing (p16^+^) CK^+^ CTCs. Given intrapatient baseline variability in CTC numbers [26], as well as the extended timeframe required for DNMT inhibitor-mediated gene expression changes [8, 27], we assessed 3 different “baseline” specimens (C1D1 pre, C1D1 post, and C1D2) and, conservatively, used the highest percentage of p16^+^CK^+^ CTCs as the “baseline” value, as described previously [8]. Also as previously established [8], p16 re-expression was defined as a ≥ 3-fold increase in the percentage of p16-expressing CTCs at any post-treatment time point compared to baseline values; for patients with 0 p16^+^CK^+^ CTCs at baseline, an increase in the proportion of p16^+^CK^+^ CTCs was defined as an increase from 0 to ≥ 2 p16^+^CK^+^ cells. Per these criteria, the majority (23 of 30; 77%) of patients evaluable for p16 CTC response demonstrated a treatment-associated increase in the proportion of p16^+^CK^+^ CTCs (Fig. 3). Of the 7 patients who did not demonstrate such an increase, 3 had high baseline levels of p16^+^CK^+^ CTCs (≥ 20%). These rates of high-baseline p16^+^CK^+^ CTC prevalence and FdCyd-induced increases in the proportion of p16^+^CK^+^ CTCs are similar to those previously observed in a phase 2 study of intravenously administered FdCyd and THU [8].

To explore concordance across tumor and blood p16 measurements and whether CK^+^ CTCs are an adequate surrogate for tumor in PD assessments of FdCyd-associated changes in p16 expression, we compared CTC p16 expression changes with treatment-induced modulation of tumor p16 expression and *CDKN2A* hypomethylation. Among the 4 patients evaluable for p16 protein expression changes in both CK^+^ CTCs and tumor biopsy specimens (patients 3010009, 3010013, 4010014, and 4010016), none showed appreciable modulation of tumor p16 expression by IHC, yet 3 had significant FdCyd-induced increases in the proportion of p16^+^CK^+^ CTCs (Table 4). In contrast, there was high concordance between FdCyd-associated changes in tumor *CDKN2A* hypomethylation and p16 expression in CK^+^ CTCs among the 6 patients evaluable for both, with 5 of 6 patients exhibiting both increased tumor *CDKN2A* hypomethylation and CK^+^ CTC p16 re-expression and the remaining patient exhibiting no increases in either p16 measurement. Together, these data suggest that p16 protein expression in circulating CK^+^ tumor cells may be more sensitive to modulation by FdCyd relative to lesion-resident tumor cells.

The proportion of CTCs expressing p16 was also measured longitudinally for vimentin-positive (V^+^), mesenchymal-like phenotype CTCs, given the potential clinical significance of this CTC population [8, 28, 29]. We evaluated p16^+^V^+^ CTCs in a pilot study, as the technology for quantitating vimentin expression became available during the course of this study. Increases in the proportion of p16-expressing V^+^ CTCs were measured in just 2 of the 22 patients evaluable for p16^+^V^+^ CTC prevalence (9%)—a substantially lower prevalence than that observed for p16 re-expression in CK^+^ CTCs and consistent with the lack of p16 expression modulation observed in tumor p16 IHC analysis (Table 4; Supplementary Fig. S7).

#### FdCyd-mediated changes in genome-wide DNA methylation

To more broadly explore FdCyd-associated changes in DNA methylation patterns, we performed bisulfite sequencing on DNA isolated from paired biopsy specimens and measured genome-wide CpG methylation to identify genomic regions that were differentially hypomethylated in response to oral FdCyd-THU therapy. The overall number of genomic regions that were significantly demethylated at C1W3 relative to baseline ranged from 123 to 1821 (median, 179), though the maximum value of 1821 for patient 1,010,020 appeared to be an outlier that was likely due to the specimens for this patient being sequenced in a later, separate batch for which the sequencing read depth was greater than that of the prior batch (Supplementary Fig. S8A). There was no obvious association between the number of demethylated regions and clinical response (stable disease vs. progressive disease) to oral FdCyd-THU therapy (Supplementary Fig. S8A).

Genes that are epigenetically regulated and have significant FdCyd-induced promoter demethylation in patients with stable disease are of particular interest, as these changes in promoter methylation status may yield transcriptional changes that are associated with FdCyd-THU activity. Therefore, we identified genes with significant treatment-induced DNA hypomethylation within 5 kilobases (kb) upstream of the transcription start site (TSS) for the 3 patients with stable disease for whom these data were available: 1,010,020 (CRC, 4 cycles SD), 4,010,016 (HNSCC, 8 cycles SD), 4,010,017 (HNSCC, 6 cycles SD). There were no genes with significant FdCyd-induced promoter hypomethylation across all 3 of these patients (Supplementary Table S8; Supplementary Fig. S8B), suggesting heterogeneity in any epigenetic modulations that may mediate clinical benefit. We focused further analyses on genes considered to be likely epigenetically regulated and identified those as genes for which RNA expression was negatively correlated with DNA promoter methylation (Pearson *r* ≤ −0.3) across GDSC (Genomics of Drug Sensitivity in Cancer) cell lines based on CellMinerCDB data and analysis tools [16]. The tumors from patients 4,010,016 and 4,010,017 each had 14 such genes with significant promoter hypomethylation in response to FdCyd-THU treatment, including several genes with known tumor suppressor function: *BMPR1B* [30], *CDH1* [31], *FCGRT* [32], *LRIG3* [33], and *MAMDC2* [34] for patient 4,010,016, and *RASSF1* [35] and *TET1* [36] for patient 4,010,017 (Table 5). Likely due to the greater sequencing read depth, patient 1,010,020 had a substantially greater number of such genes—a total of 187; these likewise included several tumor suppressor genes, such as *ANGPTL4* [37], *ARHGEF10* [38], *MARVELD1* [39], and *SQSTM1* [40]. The top most enriched molecular function gene ontology sets represented were transcription regulator activity, sequence-specific DNA binding, and DNA binding/transcription activator activity (Supplementary Table S8, Table 5). Together, these data suggest a potential role for re-expression of tumor suppressor and/or transcriptional regulator genes in mediating oral FdCyd activity.

In addition to these methylation changes within 5 kb of the TSS, we also assessed differentially methylated regions within intronic regions, as such intronic DNA methylation has been demonstrated to regulate cancer cell gene expression and alternative splicing [16, 41, 42]. As expected based on batch-specific differences in sequencing read depth, we found a substantially greater number of genes with FdCyd-induced intronic DNA hypomethylation for patient 1,010,020 compared to patients 4,010,016 and 4,010,017 (2116 vs. 311 and 307, respectively; Supplementary Fig. S8C). A total of 7 genes had significant intronic hypomethylation in response to oral FdCyd-THU in all 3 patients with stable disease, including documented tumor suppressor genes *BCYRN1* [43], *NLRP12* [44], and *RUNX1* [45] (Supplementary Fig. S8C). These data suggest a potential role of intronic hypomethylation in mediating the activity of oral FdCyd-THU therapy.

## Discussion

Oral FdCyd demonstrated a largely dose-proportional plasma PK profile, and the RP2D achieved target plasma concentrations higher than those demonstrated to inhibit DNA methylation in vitro (≥ 1 µM) [10]. As anticipated based on preclinical and pilot clinical PK studies [9], plasma C_max_ values for oral FdCyd were lower than those previously reported for the phase 1 clinical study of intravenously administered FdCyd with THU [7]. In the present study of oral FdCyd + THU, FdCyd peak concentrations at the R2PD (160 mg) were approximately 20% of those reported after the IV FdCyd R2PD of 100 mg/m^2^ [8], and comparison of previous reports of clearance (142 mL/min/m^2^) with current values (123 L/h) suggests that FdCyd bioavailability in the current study was approximately 12%—in line with the previously established 10% [9]. Similarly, THU plasma concentrations for the RP2D in the present study were approximately 9–13% lower than after administration of the intravenous RP2D of 350 mg/m^2^ THU [8]; comparing previous clearance values (43 mL/min/m^2^) with current values (142 L/h) suggests that THU bioavailability in the current study was approximately 3%, which is consistent with the previously established 4.1% [9]. Surprisingly, THU exposure was associated with DLT occurrence, while FdCyd exposure was not. While the differences in plasma THU exposure may not impact DLT via an effect on plasma FdCyd levels, intracellular increases in THU may yield more prolonged intracellular FdCyd and increased generation of phosphorylated anabolites. More detailed analyses of active intracellular moieties are needed to test this hypothesis.

In addition to modulating DNA promoter methylation, pyrimidine analog DNMT inhibitors can yield tumor DNA damage and subsequent cell cycle arrest and cell death via their conversion to 2′-deoxyuridine 5′-monophosphate (dUMP) analogs that inhibit thymidylate synthase (TS) [46]. Though THU inhibits CDA-mediated conversion of FdCyd to FdUrd (which can then be phosphorylated by uridine kinase to generate FdUMP), it does not inhibit the alternative mechanism of FdUMP formation via dCMP deaminase-mediated deamination of FdCMP (which is generated via deoxycytidine kinase-catalyzed phosphorylation of FdCyd). Therefore, it remains possible that the FdCyd-THU combination yields TS inhibition and subsequent DNA damage-related activity and/or toxicity. Indeed, the adverse event profile noted on the present study is consistent with those of TS inhibitors: namely, predominantly hematological and gastrointestinal toxicities [47]. While high-grade gastrointestinal toxicities are rare among patients treated with standard intravenous regimens of decitabine or azacytidine [48] (as well as the combination of oral decitabine and the THU analog cedazuridine [49]), diarrhea was the most common grade 3/4 toxicity, and the dose-limiting toxicity, in a phase 1 study of oral azacitidine [50], suggesting that the grade 3/4 gastrointestinal toxicities in the present study of oral FdCyd-THU are consistent with the expected class effects on TS and may also be associated with high-dose oral administration of DNMT inhibitors. While the lack of substantial pHH3 modulation measured in paired pre/on-treatment tumor biopsy specimens suggests the absence of FdCyd-induced cell cycle abnormalities in tumor, the on-treatment biopsy timepoint (C1W3) was selected for capturing sustained epigenetic effects rather than the potentially more dynamic DNA damage-related molecular changes that may occur, complicating interpretation of the pHH3 data. In light of these data, as well as the lack of clinical responses on this study, it is possible that FdCyd-THU–mediated TS inhibition may have contributed to the toxicity of this regimen in somatic tissues without providing appreciable antitumor activity, thereby narrowing the therapeutic window for this combination.

The lack of tumor regression in response to oral FdCyd + THU is consistent with the modest response rates in a prior phase 2 study of intravenously administered FdCyd + THU in patients with breast, HNSCC, urothelial transitional cell carcinoma, or NSCLC tumors (6.9%, 0%, 5.6%, and 0%, respectively) [8]. Together, these response data suggest that the FdCyd + THU combination may have limited activity when administered at tolerated doses in patients with solid tumors. Our findings in this regard may reflect a broader discrepancy between the success of DNMT inhibitors in treating myeloid malignancies and the relative paucity of clinical activity for these agents in patients with solid tumors, which may be due to the dependence of pyrimidine analog DNMT inhibitor activation on pyrimidine salvage enzymes that are more highly expressed in myeloid cells than in other tissue types. Indeed, the prevalence of hematological toxicities in this study is consistent with such differential overexpression of pyrimidine salvage enzymes in myeloid cells.

Target engagement by orally administered FdCyd, as assessed qualitatively by DNMT1 IHC, was not observed for any of the patients with assessable paired biopsies at the time of on-treatment biopsies during cycle 1 week 3. This may be explained by true absence of any FdCyd-mediated target engagement and/or by target rebound following the several-day break in dosing between the last administered dose (on cycle 1 day 13) and the time of biopsy during cycle 1 week 3 (days 14–21). The former explanation is less likely given the measured downstream DNA hypomethylation changes, and the treatment-induced increase in DNMT1 levels detected in 2 patients (including 1 patient with an on-treatment biopsy collected on C2D1) is consistent with the DNMT rebound, which has been observed for other DNMT inhibitors [22, 23].

Analyses of downstream PD effects for oral FdCyd-THU revealed treatment-associated changes that highlight potential molecular mechanisms underlying FdCyd-induced disease stabilization. FdCyd-associated promoter hypomethylation was measured for several likely epigenetically regulated genes, including tumor suppressor genes, in patients with a best response of stable disease, and it is possible that one or more of these genes mediates FdCyd antitumor activity. In examining changes in p16—a tumor suppressor and known PD biomarker of DNMT inhibitors—we measured treatment-induced increases in the number of promoter hypomethylation sites for the p16-encoding gene *CDKN2A* in tumor specimens from 7 of the 8 assessed patients. However, of the 6 biopsied patients with both tumor p16 IHC and *CDKN2A* promoter methylation data available, 5 demonstrated increases in *CDKN2A* hypomethylation, yet only 1 of these showed increased p16 expression by IHC. This discrepancy may indicate a temporal lag between *CDKN2A* promoter hypomethylation and increased p16 expression in tumor lesions or the absence of molecular components required to translate *CDKN2A* hypomethylation into increases in p16 protein. Indeed, the myriad, multi-level mechanisms of p16 regulation [51] may render FdCyd-induced changes in *CDKN2A* promoter hypomethylation insufficient for p16 re-expression at the protein level.

The proportion of patients exhibiting oral FdCyd-associated increases in p16-expressing V^+^ CTCs (9%; 2 of 22) was lower than that measured previously for intravenously administered FdCyd (50%; 6 of 12) [8], though small sample sizes preclude definitive conclusions regarding this difference. Among the biopsied patients with CTC data, this paucity of p16 response in the V^+^ CTC population was consistent with the lack of qualitative tumor p16 modulation as detected by IHC at C1W3, suggesting that changes (or lack thereof) in p16 protein expression in V^+^ CTCs may reflect those in tumor tissue. In addition, the high prevalence of treatment-induced increases in p16-expressing CTCs measured in the CK^+^ CTC population was not consistent with the low prevalence of tumor p16 expression increases measured by IHC within the same timeframe, suggesting that CK^+^ tumor cells in circulation may be more sensitive to FdCyd-induced p16 changes than cells within tumor lesions or that the kinetics of p16 expression changes differ between tumor cells in circulation versus those within a lesion; regardless, these results indicate that CTC p16 measurements may not be an adequate surrogate for tumor changes in p16 expression at the timepoints examined in this study.

## Supplementary Information

Below is the link to the electronic supplementary material.

Supplementary Material 1

Supplementary Material 2

## Data Availability

All data are provided within the manuscript or supplementary information files.
