# Supplementary material for: A phase 1 study of orally administered 5-fluoro-2’-deoxycytidine with tetrahydrouridine in patients with refractory solid tumors
Source: Cancer Chemother Pharmacol. 2025 Dec 22;96(1):1. doi: 10.1007/s00280-025-04844-y (PMC12722308; doi:10.1007/s00280-025-04844-y)
Supplement: Supplementary file 1 — Supplementary Material 1 [file 280_2025_4844_MOESM1_ESM.docx]

# Supplementary Tables

## Supplementary Table S1. Dose escalation schema for the orally administered FdCyd-THU combination.

| **Dose level** | **FdCyd**  **(mg)** | **THU**  **(mg)** |
| --- | --- | --- |
| 1 | 30 mg QD | 3000 QD days 1-3 week 1, days 8-10 week 2, week 3 off |
| 2 | 60 mg QD | 3000 QD days 1-3 week 1, days 8-10 week 2, week 3 off |
| 3 | 90 mg QD | 3000 QD days 1-3 week 1, days 8-10 week 2, week 3 off |
| 4 | 120 mg QD | 3000 QD days 1-3 week 1, days 8-10 week 2, week 3 off |
| 5 | 160 mg QD | 3000 QD days 1-3 week 1, days 8-10 week 2, week 3 off |
| 6 | 160 mg QD | 3000 QD days 1-4 week 1, days 8-11 week 2, week 3 off |
| 7 | 160 mg QD | 3000 QD days 1-5 week 1, days 8-12 week 2, week 3 off |
| 8** | 160 mg QD | 3000 QD days 1-6 week 1, days 8-13 week 2, week 3 off |
| 9 | 160 mg QD | 3000 QD days 1-7 week 1, days 8-14 week 2, week 3 off |
| 10 | 60 mg BID | 6000 BID days 1-5 week 1, days 8-12 week 2, week 3 off |
| 10A* | 40 mg BID | 3000 BID days 1-5 week 1, days 8-12 week 2, week 3 off |
| 11 | 80 mg BID | 6000 BID days 1-5 week 1, days 8-12 week 2, week 3 off |

FdCyd and THU were administered orally in 21-day cycles.

BID, twice daily; QD, once daily.

*Patients requiring dose reduction from DL10 were de-escalated to DL10A.

**MTD/RP2D/expansion phase dose

## Supplementary Table S2. Dose-limiting toxicities for oral FdCyd-THU.

| **Dose level** | **No. pts enrolled** | **No. pts with DLT** | **DLT(s)** |
| --- | --- | --- | --- |
| **1** | 3 | — | — |
| **2** | 4 | — | — |
| **3** | 3 | — | — |
| **4** | 3 | — | — |
| **5** | 3 | — | — |
| **6** | 3 | — | — |
| **7** | 3 | — | — |
| **8** | 24 | — | — |
| **9** | 2 | 2 | Gr3 refractory nausea, vomiting, and diarrhea; Gr3 diarrhea |
| **10** | 7 | 2 | Gr3 oral mucositis; Gr4 neutropenia |
| **10A** | 1 | — | — |
| **11** | 3 | 2 | Gr3 nausea, vomiting, and diarrhea;  Gr4 thrombocytopenia |

**^†^**Maximum tolerated dose and recommended phase 2 dose

Gr3, grade 3; Gr4, grade 4

## Supplementary Table S3. FdCyd plasma pharmacokinetic parameters after oral administration of FdCyd and THU.

| **Dose level** | **FdCyd,THU doses**  **(mg)** | **C_max_**  **(mg/L)** | **C_max_/Dose**  **(µg/L / mg)** | **T_max_**  **(h)** | **t½**  **(h)** | **AUC_0-inf_^a^**  **(mg/L•h)** | **Cl/F**  **(L/h)** | **V_z_/F^b^**  **(L)** | **FdCyd_2h-D1_**  **(mg/L)** | **FdCyd_2h-D2_**  **(mg/L)** | **FdCyd_2h-D3_**  **(mg/L)** |
| --- | --- | --- | --- | --- | --- | --- | --- | --- | --- | --- | --- |
| DL1 | 30, 3000 (*n*=3) | 0.09 (2.3) | 3.0 (2.3) | 0.9 (2.5) | 0.6 (1.1) | 0.14 (2.77) | 212 (2.77) | 187 (2.6) | 0.44 (2.20) | 0.076 (3.80) | 0.042 (2.6) |
| DL2 | 60, 3000 (*n*=4) | 0.29 (3.0) | 4.9 (3.0) | 1.0 (2.3) | 0.7 (1.6) | 0.63 (3.08) | 95.7 (3.08) | 90 (4.5) | 0.203 (3.87) | 0.169 (3.80) | 0.145 (1.14) |
| DL3 | 90, 3000 (*n*=3) | 0.53 (1.8) | 5.8 (1.8) | 1.5 (1.0) | 1.1 (1.5) | 1.06 (1.91) | 84.7 (1.91) | 139 (2.0) | 0.525 (1.79) | 0.449 (1.55) | 0.460 (2.89) |
| DL4 | 120, 3000 (*n*=3) | 0.89 (2.0) | 7.4 (2.0) | 1.2 (2.3) | 0.6 (1.2) | 1.59 (2.43) | 75.6 (2.43) | 62 (2.0) | 0.611 (3.17) | 0.499 (9.47) | 0.891 (2.08) |
| DL5-9 | 160, 3000 (*n*=31) | 0.58 (2.1) | 3.6 (2.1) | 1.4 (1.6) | 0.6 (1.5) | 1.20 (2.35) | 133 (2.35) | 114 (2.2) | 0.505 (1.92) | 0.595 (3.29) | 0.650 (1.98) |
|  | *All, 3000 (n=44)* | *-* | *4.0 (2.2)^b^* | *1.3 (1.7)* | *0.6 (1.5)* | *-* | *123 (2.37)^c^* | *111 (2.4)* | *-* | *-* | *-* |
| DL10 | 60, 6000 (*n*=8) | 0.38 (1.6) | 6.4 (1.6) | 0.8 (1.8) | 0.5 (1.3) | 0.63 (1.71) | 96.0 (1.71) | 63 (1.8) | 0.317 (1.98) | 0.559 (1.63) | 0.584 (1.60) |
| DL11 | 80, 6000 (*n*=3) | 0.57 (1.6) | 7.1 (1.6) | 1.5 (1.0) | 0.5 (1.3) | 1.22 (1.41) | 65.8 (1.41) | 44 (1.5) | 0.571 (1.64) | 0.931 (1.99) | 0.745 (2.48) |
|  | *All, 6000 (n=11)* | *-* | *6.6 (1.6)^b^* | *0.9 (1.8)* | *0.5 (1.3)* | *-* | *85.7 (1.65)^c^* | *56 (1.7)* | *-* | *-* | *-* |
|  | **Total (*n*=55)** | - | 4.4 (2.1) | 1.2 (1.8) | 0.6 (1.5) | - | 115 (2.26) | 98 (2.3) | - | - | - |

Geometric mean (geometric standard deviation) values are shown for each parameter.

^a^AUC_0-inf_ extrapolated beyond the last time point sampled was geometric mean 1.4% (range 0.2-25%).

^b^P=0.028 by Wilcoxon rank test.

^c^P=0.16 by Wilcoxon rank test.

## Supplementary Table S4. FdUrd and FU plasma pharmacokinetic parameters and FdUrd/FdCyd metabolic ratios after oral administration of FdCyd and THU.

| **Dose level** | **FdCyd,THU doses**  **(mg)** | **FdUrd** | | | | | **FdUrd/FdCyd** | | **FU** | | | |
| --- | --- | --- | --- | --- | --- | --- | --- | --- | --- | --- | --- | --- |
|  |  | C_max_  (mg/L) | C_max_/Dose  (µg/L / mg) | AUC_0-inf_^a^  (mg/L•h) | AUC/Dose  (µg/L•h/mg) | t½  (h) | C_max_ | AUC_0-inf_ |  | C_max_  (mg/L) | | AUC_0-inf_^b^  (mg/L•h) |
| DL1 | 30, 3000 (*n*=3) | 0.012 (1.4) | 0.41 (1.4) | 0.047 (-) | 1.58 (-) | 3.3 (-) | 0.099 (1.6) | 0.069 (-) | *n*=0 | - | | - |
| DL2 | 60, 3000 (*n*=4) | 0.011 (1.7) | 0.19 (1.7) | 0.044 (1.72) | 0.73 (1.7) | 1.8 (3.4) | 0.039 (2.3) | 0.070 (3.5) | *n*=3 | 0.0042 (1.2) | | 0.019 (1.70) |
| DL3 | 90, 3000 (*n*=3) | 0.015 (1.2) | 0.16 (1.2) | 0.043 (1.20) | 0.48 (1.2) | 1.1 (1.3) | 0.028 (1.7) | 0.041 (1.7) | *n*=3 | 0.0055 (1.1) | | 0.026 (1.54) |
| DL4 | 120, 3000 (*n*=3) | 0.016 (1.6) | 0.14 (1.6) | 0.054 (1.97) | 0.45 (2.0) | 1.7 (1.2) | 0.018 (1.3) | 0.034 (1.3) | *n*=2 | 0.0082 (1.4) | | 0.032 (1.12) |
| DL5-9 | 160, 3000 (*n*=31) | 0.031 (1.5) | 0.20 (1.5) | 0.084 (1.55) | 0.53 (1.6) | 1.2 (1.7) | 0.054 (1.9) | 0.070 (1.8) | *n*=29 | 0.015 (1.6) | | 0.052 (1.66) |
|  | *All, 3000 (n=44)* | *-* | *0.19 (1.6)^c^* | *-* | *0.55 (1.6)^d^* | *1.3 (1.9)* | *0.048 (2.0)^e^* | *0.068 (2.1)^f^* | *-* | *-* | | *-* |
| DL10 | 60, 6000 (*n*=8) | 0.023 (1.4) | 0.38 (1.4) | 0.047 (1.59) | 0.79 (1.6) | 1.0 (1.5) | 0.060 (1.8) | 0.072 (1.4) | *n*=8 | 0.011 (1.3) | | 0.033 (1.30) |
| DL11 | 80, 6000 (*n*=3) | 0.019 (1.6) | 0.24 (1.6) | 0.068 (1.68) | 0.85 (1.7) | 1.7 (1.5) | 0.033 (1.8) | 0.056 (1.2) | *n*=3 | 0.010 (1.1) | | 0.044 (1.36) |
|  | *All, 6000 (n=11)* | *-* | *0.34 (1.5)^c^* | *-* | *0.80 (1.6)^d^* | *1.2 (1.6)* | *0.051 (1.9)^e^* | *0.067 (1.4)^f^* | *-* | *-* | *-* | |
|  | **Total (*n*=55)** | - | 0.22 (1.6) | - |  | 1.3 (1.8) | - | - | - | - | - | |

Geometric mean (geometric standard deviation) values are shown for each parameter.

^a^AUC_0-inf_ extrapolated beyond the last time point sampled was geometric mean 18% (range 3.9-89.3%).

^b^AUC_0-inf_ extrapolated beyond the last time point sampled was geometric mean 34% (range 11-84%).

^c^P=0.0016 by Wilcoxon rank test.

^d^P=0.028 by Wilcoxon rank test.

^e^P=0.93 by Wilcoxon rank test.

^f^P=0.82 by Wilcoxon rank test.

## Supplementary Table S5. THU plasma pharmacokinetic parameters after oral administration of FdCyd and THU.

| **Dose**  **level** | **Dose**  **(mg)** | **C_max_**  **(mg/L)** | **C_max_/Dose**  **(µg/L / g)** | **T_max_**  **(h)** | **t½**  **(h)** | **AUC_0-6_**  **(mg/L•h)** | **AUC_0-6_/Dose**  **(µg/L•h / mg)** | **AUC_0-inf_^a^**  **(mg/L•h)** | **Cl/F**  **(L/h)** | **V_z_/F**  **(L)** | **C_min24h_**  **(mg/L)** | **C_min48h_**  **(mg/L)** |
| --- | --- | --- | --- | --- | --- | --- | --- | --- | --- | --- | --- | --- |
| DL1-DL9 | 3000 (*n*=44) | 1.52 (1.65) | 505 (1.7) | 3.0 (1.6) | 8.8 (1.6) | 6.75 (1.63) | 2.25 (1.63) | 21.1 (1.8) | 142 (1.8) | 1809 (1.7) | 0.26 (1.86) | 0.33 (2.09) |
| DL10-DL11 | 6000 (*n*=11) | 2.34 (1.40) | 390 (1.4) | 3.2 (1.6) | 7.4 (1.4) | 10.8 (1.37) | 1.79 (1.37) | 31.6 (1.6) | 190 (1.6) | 2037 (1.6) | 2.52 (2.01) | 3.17 (1.76) |
|  | **Total (*n*=55)** | - | 480 (1.6) | 3.0 (1.6) | 8.7 (1.6) | - | 2.15 (1.60) | - | 147 (1.7) | 1831 (1.7) | - | - |

Geometric mean (geometric standard deviation) values are shown for each parameter. Half-life values for only 6 patients could be determined at 6000 mg.

^a^AUC_0-inf_ extrapolated beyond the last time point sampled was geometric mean 24% (range 7.1-76%) for 3000 mg and 63% (range 53-77%) for 6000 mg profiles.

## Supplementary Table S6. Urinary excretion of FdCyd, FdUrd, FU, and THU over 0-24 hours of day 1.

| **Dose level** | **FdCyd, THU doses**  **(mg)** | **FdCyd**  **(% dose)** | ***N*** | **FdUrd**  **(% dose)** | ***N*** | **FU**  **(% dose)** | ***N*** | **THU**  **(% dose)** | ***N*** |
| --- | --- | --- | --- | --- | --- | --- | --- | --- | --- |
| DL1 | 30, 3000 (*n*=3) | - | - | - | - | 0.20 (-) | 1 | 2.9 (2.0) | 3 |
| DL2 | 60, 3000 (*n*=4) | - | - | - | - | 0.51 (-) | 1 | 2.8 (1.6) | 3 |
| DL3 | 90, 3000 (*n*=3) | - | - | - | - | - | - | 2.1 (1.4) | 3 |
| DL4 | 120, 3000 (*n*=3) | - | - | 0.064 (1.0) | 2 | 0.34 (-) | 1 | 1.1 (4.8) | 3 |
| DL5-9 | 160, 3000 (*n*=31) | 0.083 (2.2) | 6 | 0.047 (1.3) | 3 | 0.38 (2.1) | 24 | 2.1 (1.8) | 30 |
|  | *All, 3000 (n=44)* | *0.083 (2.2)* | *6* | *0.053 (1.3)* | *5* | *0.37 (2.0)* | *27* | *2.1 (2.0)* | *42* |
| DL10 | 60, 6000 (*n*=8) | 0.15 (3.2) | 2 | 0.14 (2.6) | 2 | 0.32 (2.5) | 6 | 1.1 (2.9) | 7 |
| DL11 | 80, 6000 (*n*=3) | 0.058 (-) | 1 | 0.045 (-) | 1 | 0.21 (3.0) | 3 | 1.6 (1.7) | 3 |
|  | *All-6000 (n=11)* | *0.11 (2.6)* | *3* | *0.10 (2.6)* | *3* | *0.27 (2.5)* | *9* | *1.2 (2.5)* | *10* |
|  | **Total (*n*=55)** | - |  | - |  | - |  | 1.9 (2.1) | 52 |

Geometric mean (geometric standard deviation) values are shown for each parameter.

## Supplementary Table S7. Qualitative IHC analysis of tumor DNMT1 levels.

| **Patient** | **Diagnosis** | **Best response**  **(Cycles)** | **DNMT1 IHC** | |
| --- | --- | --- | --- | --- |
|  |  |  | **C1D1** | **C1W3** |
| 3010009* | Pancreatic | PD (2) | –/+ | –/+ |
| 4010014 | Esophageal | PD (1) | +++ | +++ |
| 4010016 | Head & Neck | SD (8) | –/+ | –/+ |
| 4010017 | Head & Neck | SD (6) | –/+ | NA |
| 3010013 | Colorectal | PD (2) | –/++ | –/++ |
| 5010001 | Colorectal | PD (2) | –/++ | +++ |
| 1010019 | Breast | NP (1) | –/++ | –/++ |
| 1010020 | Colorectal | SD (4) | –/+ | ++ |

IHC qualitative scoring showing DNMT1 levels ranging from low to high is indicated by shading intensity (light = low; dark = high) and as follows: –/+, –/++, ++, and +++.

*The on-treatment biopsy for patient 3010009 was collected during cycle 2 week 3 (C2W3).

## Supplementary Table S8. All genes with significant FdCyd-THU–induced promoter hypomethylation in patients with a best response of stable disease. *Excel file only*.

# Supplementary Figures


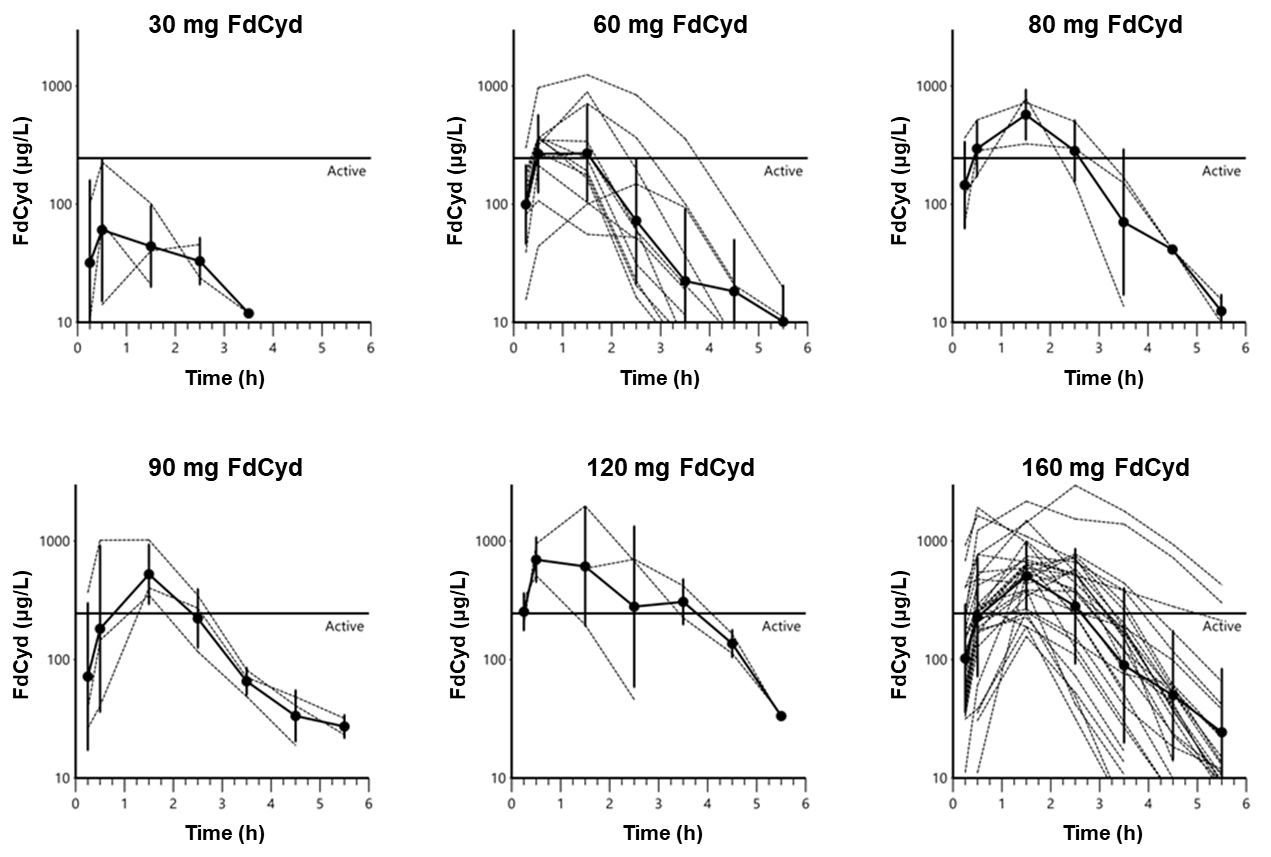


## Supplementary Figure S1. Plasma pharmacokinetic profiles of orally administered FdCyd. Concentration🞨time profiles for the indicated FdCyd doses are shown for individual patients (dashed lines), along with geometric means (solid lines and circles); error bars indicate geometric standard deviations. Active target exposure of 245 ng/mL (1 μM) is indicated by thick horizontal black lines.


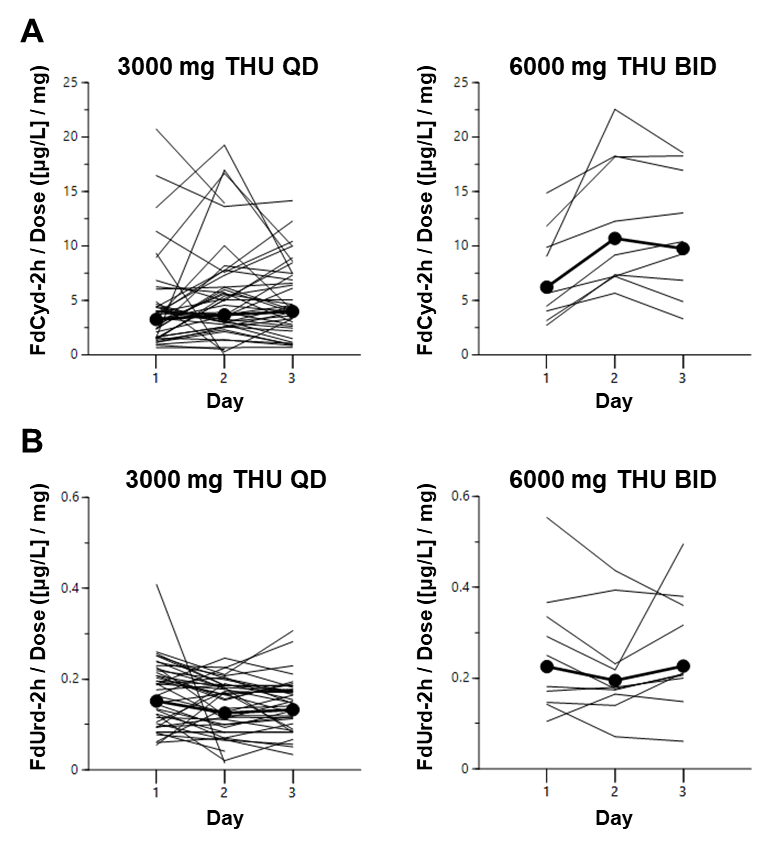


## Supplementary Figure S2. Dose-normalized plasma concentrations of FdCyd and FdUrd following oral FdCyd-THU administration. Plasma concentrations of FdCyd (A, B) or FdUrd (C, D) are shown for patients receiving 3000 mg once-daily THU (left) or 6000 mg twice-daily THU (right). Thin lines represent values for individual patients, while thick lines and circles represent geometric means.


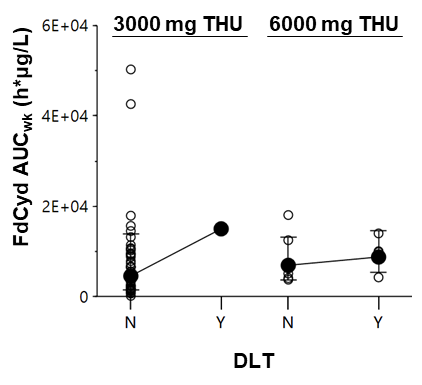


## Supplementary Figure S3. FdCyd exposure-toxicity analysis following oral administration of FdCyd-THU. Week 1 FdCyd AUC is based on day 1 AUC_inf_, days of dosing, and dose schedule for patients receiving 3000 mg QD THU or 6000 mg BID THU. Individual patients (open circles) and geometric means (solid circle) and standard deviations (solid line) are shown.


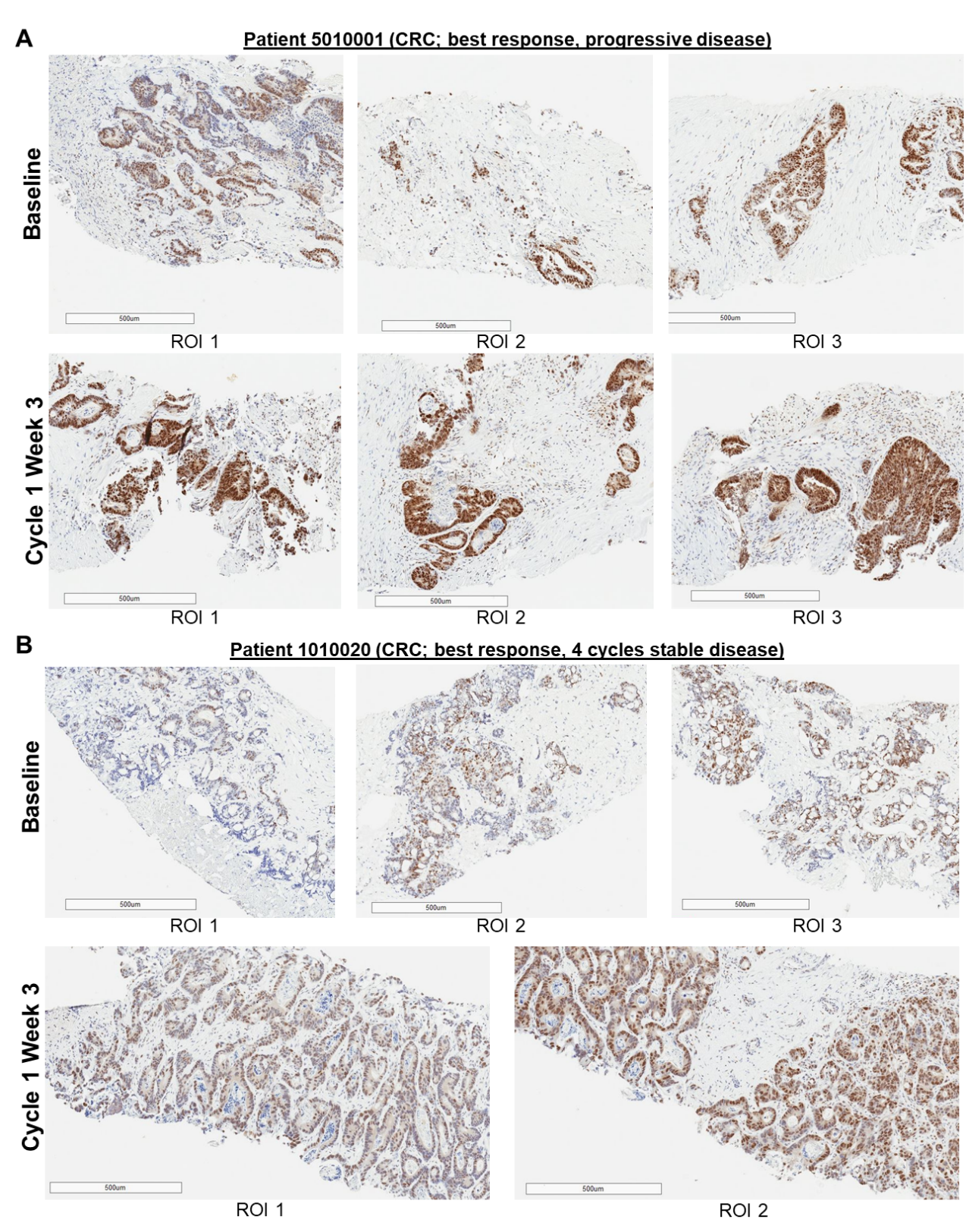


## Supplementary Figure S4. Select images from DNMT1 IHC analysis of tumor biopsy specimens collected at baseline and following FdCyd-THU administration. Multiple regions of interest (ROI) showing DNMT1 IHC staining for pre-dose (Cycle 1 Day 1) and on-treatment (Cycle 1 Week 3) tumor biopsy specimens from 2 patients with colorectal carcinoma (CRC): patient 5010001 (A) and patient 1010020 (B). Scale bars indicate 500 μm.


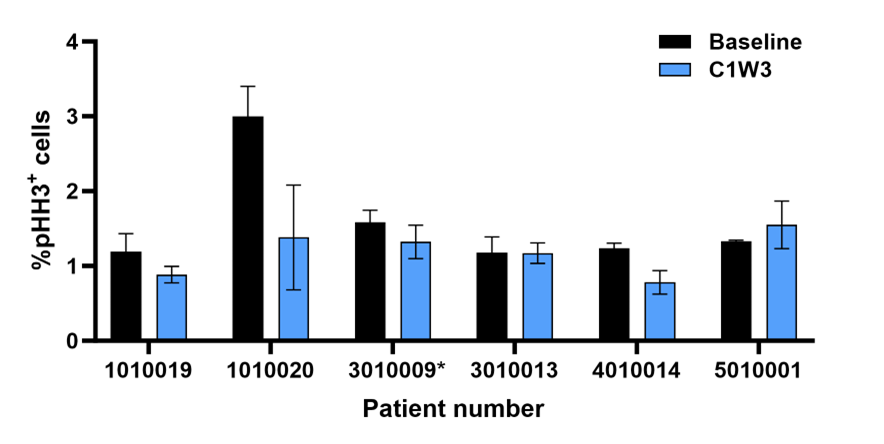


## **Supplementary Figure S5.** Quantitative immunofluorescence microscopy analysis of the mitotic marker serine 10–phosphorylated histone H3 in tumor biopsy specimens collected at baseline and following FdCyd-THU administration. Immunofluorescence staining for pHH3 and quantitation of the percentage of tumor cells expressing pHH3 were performed as described previously [[1](#_ENREF_1)]. For each timepoint, pHH3 was measured in a minimum of 4,700 nuclei per patient tumor; values shown indicate mean %pHH3^+^ tumor cells, and error bars indicate standard deviation between 2 different tumor biopsy cores per patient (for patients 1010019, 1010020, 3010009, and 5010001) or between 2 different regions of interest within a single tumor biopsy core (patients 3010013 and 4010014), depending on tumor biopsy core availability. Measurements were made in cores collected at baseline (cycle 1 day 1, pre-dose; black) or on cycle 1 week 3 (C1W3) after the start of FdCyd-THU treatment, with the exception of patient 3010009 (*), for whom the on-treatment biopsy was collected during cycle 2 week 3 (C2W3).


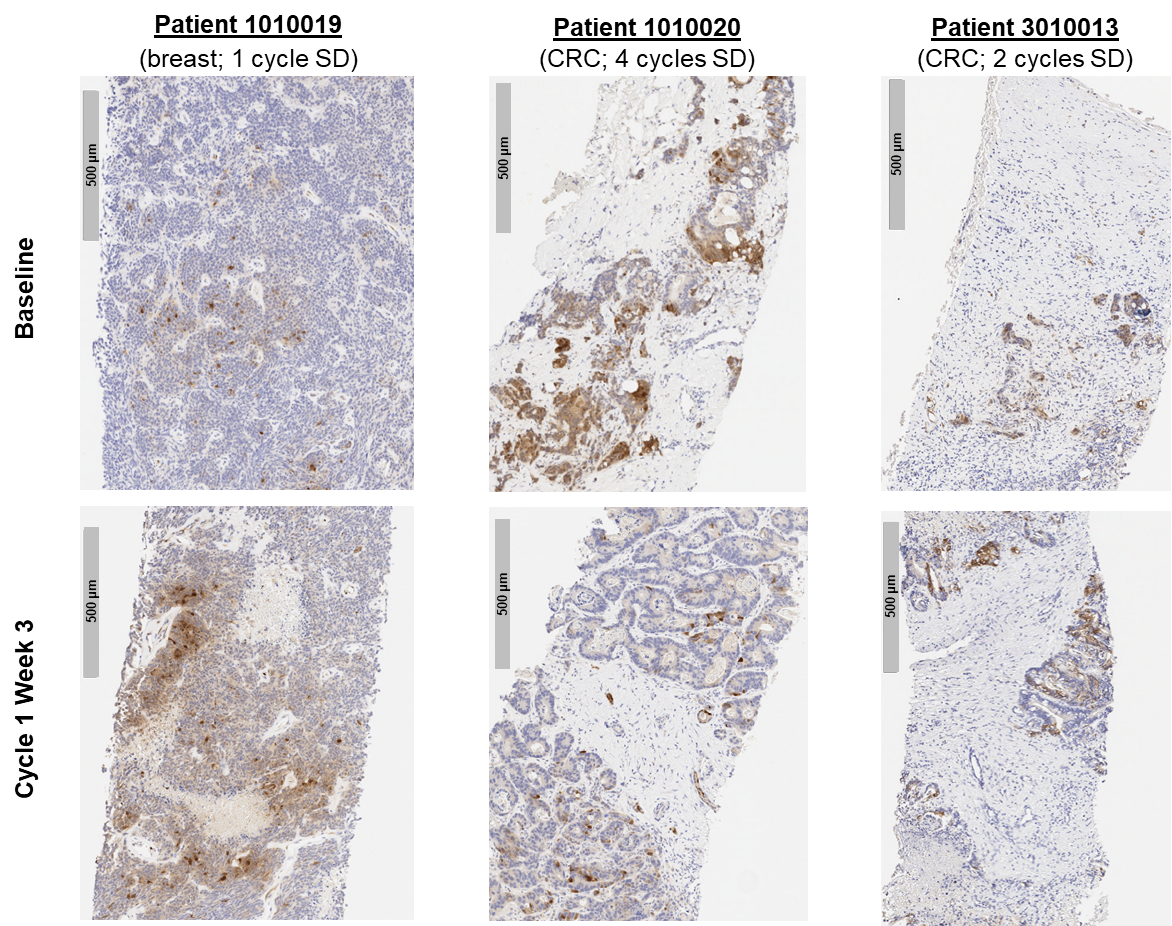


**Supplementary Figure S6**. Pharmacodynamic analysis of p16 expression in tumor biopsy specimens and cytokeratin-positive circulating tumor cells from patients treated with oral FdCyd combined with THU. Representative images from tumor p16 IHC analysis for patients 1010019, 1010020, and 3010013 at baseline (top) and cycle 1 week 3 (bottom). Patient diagnosis and best response to oral FdCyd + THU are shown. Scale bars indicate 500 μm.


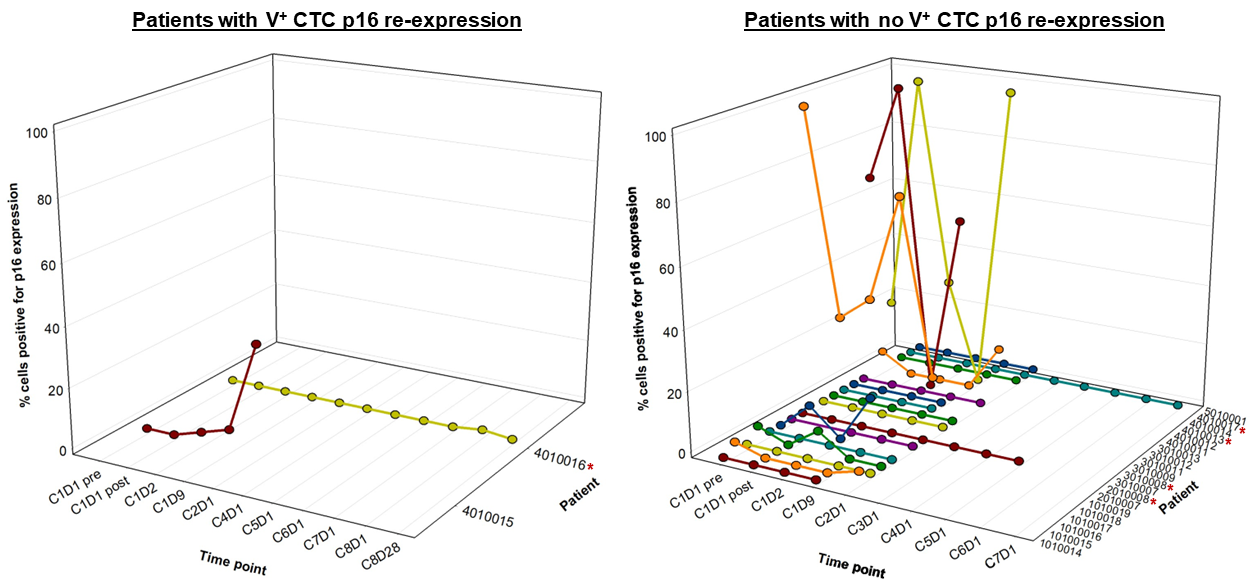


## Supplementary Figure S7. P16 re-expression in vimentin-positive circulating tumor cells following oral FdCyd-THU administration. The % V^+^ CTCs positive for p16 over time is shown for patients in which FdCyd-induced CTC p16 re-expression was measured (left; *n* = 2) and those for whom no such CTC p16 re-expression was observed (right; *n*= 16). Red asterisks indicate patients with a best response of stable disease.


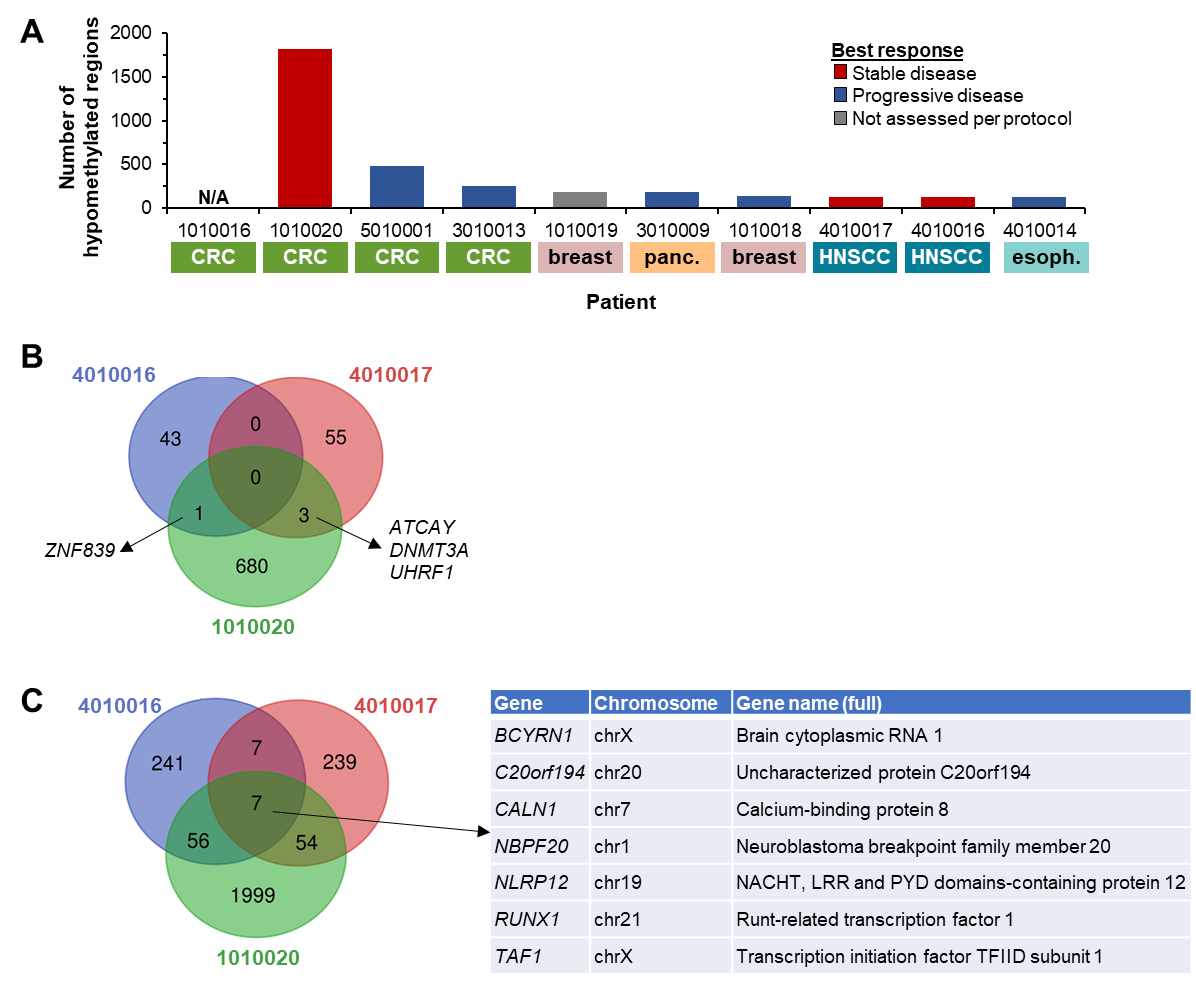


## Supplementary Figure S8. Oral FdCyd-THU–induced changes in genome-wide DNA methylation. A) The number of genomic regions that are hypomethylated in on-treatment (C1W3) relative to baseline (C1D1 pre-dose) tumor biopsy specimens is not substantially associated with tumor type or best response to FdCyd-THU therapy. B and C) Genes with differentially hypomethylated promoter regions (B) or intronic regions (C) in biopsied patients with a best response of stable disease. The numbers of genes with differential hypomethylation unique to each SD patient (1010020, 4010016, and 4010017), as well as those shared between 2 or more patients, are indicated in Venn diagrams. In panel C, the 7 genes with differential intron hypomethylation in all 3 patients with SD are listed on the right.

# Supplementary References

1. Wilsker DF, Barrett AM, Dull AB, Lawrence SM, Hollingshead MG, et al. (2019) Evaluation of Pharmacodynamic Responses to Cancer Therapeutic Agents Using DNA Damage Markers*.* Clin Cancer Res 25: 3084-3095. <https://doi.org/10.1158/1078-0432.Ccr-18-2523>.
